# Membrane-Free Stem Cells and Pyridoxal 5′-Phosphate Synergistically Enhance Cognitive Function in Alzheimer’s Disease Mouse Model

**DOI:** 10.3390/antiox11030601

**Published:** 2022-03-21

**Authors:** Ji Myung Choi, Hye Sook Park, Mei Tong He, Young Sil Kim, Hyun Young Kim, Ah Young Lee, Eun Ju Cho

**Affiliations:** 1 Department of Food Science and Nutrition, Pusan National University, Busan 46241, Korea; poutia@naver.com (J.M.C.); candol1226@naver.com (H.S.P.); skyham16@gmail.com (M.T.H.); 2 Department of Food Science and Biotechnology, Kyungsung University, Busan 48434, Korea; 3 T-Stem Co., Ltd., Changwon 51573, Korea; tstem@t-stem.com; 4 Department of Food Science and Nutrition, Gyeongsang National University, Jinju 52828, Korea; hyunyoung.kim@gnu.ac.kr

**Keywords:** membrane-free stem cell, pyridoxal 5′-phosphate, amyloid beta, Alzheimer’s disease, cognitive ability

## Abstract

Accumulation of amyloid beta (Aβ) is a major pathological hallmark of Alzheimer’s disease (AD). In this study, we evaluated the protective effect of membrane-free stem cell extract (MFSCE), which is a component of adipose-tissue-derived stem cells, on cognitive impairment in Aβ_25–35_-injected AD mice. The ICR mice were i.c.v. injected with Aβ_25–35_ and then treated with MFSCE for 14 days (i.p.). The Aβ_25–35_-injected mice showed deficits in spatial and object perception abilities, whereas treatment with MFSCE inhibited Aβ_25–35_-induced learning and memory impairment in the T-maze, novel object recognition, and Morris water maze tests. Moreover, Aβ_25–35_-induced lipid peroxidation and nitric oxide overproduction were attenuated by treatment with MFSCE. These antioxidant effects of MFSCE were related to the inhibition of the apoptotic signaling pathway. In particular, the combination treatment of MFSCE and pyridoxal 5′-phosphate (PLP) showed greater suppression of Bax and cleaved caspase-3 protein expression compared to the MFSCE- or PLP-only treatment. Furthermore, the MFSCE and PLP combination significantly downregulated the amyloidogenic-pathway-related protein expressions, such as amyloid precursor protein, presenilin 1, and presenilin 2. Therefore, the MFSCE and PLP combination may synergistically prevent Aβ_25–35_-induced neuronal apoptosis and amyloidogenesis, which contributes to cognitive improvement and has potential therapeutic implications for AD patients.

## 1. Introduction

Alzheimer’s disease (AD), a common disorder among older adults (age >65 years), is characterized by a decline in learning and memory impairment. Neuropathological features of AD are complex and include neurofibrillary tangles, hyperphosphorylated tau protein, and synaptic loss [1,2]. In particular, the accumulation of amyloid beta (Aβ) is considered a crucial marker of AD [3]. Aβ is a 39–43 amino acid peptide that is derived from the cleavage of amyloid precursor protein (APP) by β- and γ-secretase. β-secretase cleaves APP and generates a C-terminal fragment (CTF-β), which is subsequently cleaved by γ-secretase to generate Aβ [4]. Aβ fibrilization and deposition in the brain triggers pathological events, such as neuroinflammation and oxidative stress, which induces the overproduction of proinflammatory cytokines and reactive oxygen species (ROS) [5,6].

Stem cells have widespread application, including inflammatory, cardiovascular, and neurological disease. Stem cells are self-renewing multipotent cells that differentiate into several cell types, such as osteoblasts, adipocytes, or even neuronal cells, under specific conditions [7,8]. However, the therapeutic use of stem cells is hindered by several problems, including potential tumor progression, low efficiency of engraftment, immune responses, and difficulty in preadministration quality control [9]. Thus, non-cell-based therapeutics constitute an alternative approach to overcome the limitations of cell-based therapy. Membrane-free stem cell extract (MFSCE) is obtained from adult stem cells of adipose tissue following membrane removal using a patented technology [10]. MFSCE is one of the most promising stem cell therapies in use because of its greater stability during storage and nonimmunogenicity. In addition, MFSCE exerts an anti-inflammatory effect via the suppression of nitric oxide (NO) production in RAW 264.7 macrophage cells. We previously demonstrated that MFSCE exerted a protective effect against 3-morpholinosydnonimine (SIN-1)-induced oxidative stress in LLC-PK_1_ cells [11]. Furthermore, through downregulation of nuclear factor-κB and caspase activation, MFSCE protected against hydrogen-peroxide-induced periodontal inflammatory responses [12]. MFSCE also significantly suppressed Aβ-induced neuroinflammation, apoptosis, and amyloidogenesis-related protein expressions in SH-SY5Y neuronal cells [13]. However, the in vivo protective effect of MPSCE against Aβ_25–35_-induced cognitive impairment has not been studied yet.

Vitamins play an important role in cell survival, proliferation, and differentiation. In addition, pyridoxal 5′-phosphate (PLP), a dominant active form of vitamin B_6_, biologically functions as a cofactor for many enzymatic reactions, including glucose, protein, and lipid metabolisms [14,15]. Vitamin B_6_ plays important roles in the biosynthesis of DNA and cell organelles for cell growth and proliferation [16]. In addition, PLP has been involved in stabilization of protein and neurotransmitter biosynthesis [17]. Therefore, we hypothesized that the MFSCE–PLP complex possibly contributes to MFSCE metabolism and enhances cellular uptake in the brain.

This study was designed to elucidate the protective effect of MFSCE against Aβ_25–35_-induced cognitive dysfunction as assessed by behavioral tasks and underlying mechanism of MFSCE on cognitive improvement, which was determined by analyzing apoptosis- and amyloidogenesis-related markers in the mice brain. Furthermore, this study investigated whether the combination of MFSCE and PLP potentiates the neuroprotective effect against Aβ.

## 2. Materials and Methods

### 2.1. Preparation of MFSCE

The MFSCE used in this study was produced by T-Stem Co. (Changwon, Republic of Korea) as previously reported [10]. Human fatty tissue was obtained from a healthy female in her twenties with 2-degree obesity (BMI 25–29.9), which was proven to be safe based on blood tests. The donor’s consent was approved by the Regional Committee on Biomedical Research Ethics. Isolated fatty tissues were purified, and the extracted cells were cultured using serum-free cell culture medium at 37 °C, 5% CO_2_ incubator. Cells were subcultured until confluence (70–80% until 6–8 passages). Then, stem cells were collected, and the cell membranes were removed by ultrasonication. The MFSCE was further lyophilized and stored. The final MFSC product was approved by the Good Laboratory Practice (GLP) accreditation authority.

### 2.2. Animals and Experimental Protocols

The ICR mice (male, 5 weeks old, weighing 25–27 g; Orient Inc. Seongnam, Republic of Korea) were housed in plastic cages and maintained in a controlled environment (temperature 20 ± 2 °C, humidity 50 ± 10%, 12 h light/dark cycle) with ad libitum standard chow and water. Mice were divided into six groups comprising 9–10 mice each, as defined in Table 1. At baseline, there were no significant intergroup differences in the initial body weights. Animal experiments were conducted following the Guidelines for Care and Use of Laboratory Animals published by the Pusan National University Institutional Animal Core and Use Committee (Approval Number: PNU-2020-2687).

### 2.3. Aβ_25–35_-Infused Mouse Model

**Figure 1:**
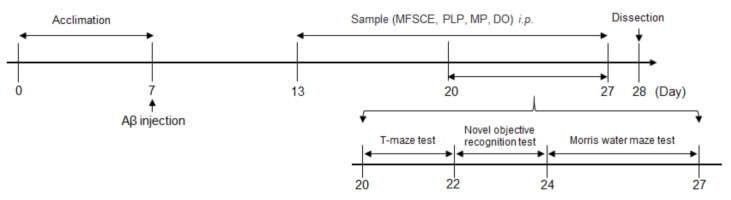
Experimental and behavioral procedure for mice.

Aβ_25–35_ peptide (Sigma Aldrich, St. Louis, MO, USA) was aggregated as previously described by Maurice et al. [18]. The Aβ_25–35_ peptide was dissolved in saline solution (0.9% NaCl) to a concentration of 1 mg/mL and incubated at 37 °C for 3 days to induce aggregation. Aβ_25–35_ was injected into the mice brain as described by Laursen and Belknap [19]. Mice were lightly anesthetized with zoletil50 (Virbac Korea, Seoul, Korea) and rompun (Bayer Korea, Seoul, Korea) mixture by intraperitoneal (i.p.) injection, and the aggregated Aβ_25–35_ was injected precisely 0.8 mm posterior to the bregma and 1.5 mm lateral to the sagittal suture. All injections were administered via a 26-gauge needle that was inserted 2.2 mm beneath from the surface of the brain using a microinfusion pump (Boading Longer Precision Pump Co., Ltd., Baoding, China). Animals were injected with 5 μL saline solution (0.9% NaCl) or 5 nmol Aβ_25–35_ in the cerebral lateral ventricle at a flow rate of 1 μL/min. The behavioral experimental schedule for mice injected with Aβ_25–35_ is shown in Figure 1.

### 2.4. Behavioral Tests

#### 2.4.1. T-Maze Test

A T-maze test was conducted using a procedure that was previously established by Montgomery [20]. The T-maze apparatus comprised a start box, left arm, and right arm with a door to separate the two arms (length of start and goal stems = 50 cm, width = 13 cm, height = 20 cm). On the day prior to T-maze tests, each mouse was placed in the start position and allowed to freely explore the arena for 10 min (habituation session). In the training session, the mice were placed at the start box and allowed to explore only the right arm for 10 min, and the number of explorations on the right arm was recorded. Then, 24 h after the training session, the mice were allowed to explore both the right and left arms of the T-maze freely for 10 min, and the number of explorations in each arm was recorded (test session) and the space perception ability (%) was calculated as the ratio of the number of left or right maze entries to the number of total maze entries multiplied by 100.

#### 2.4.2. NOR Test

The NOR test was performed according to the method described by Bevins and Besheer [21]. On the day prior to novel object recognition tests, each mouse was placed in a square field and allowed to freely explore the arena for 10 min (habituation session). In the training session, two identical objects (A, A’) were placed at fixed distances within the black square field. The mice were placed at the center of the square field, and the number of touches of each object was recorded for 10 min. Then, 24 h after the training session, one of the familiar objects was replaced with another object (A, B). The mice were then allowed to freely explore the field for 10 min, and the number of touches was recorded. The object perception ability (%) was calculated as the ratio of the number of touches to any one of the two original objects (training session) or the novel object (test session) multiplied by 100.

#### 2.4.3. MWM Test

The MWM test was conducted as described by Morris [22] with slight modifications. The apparatus included a dark plastic circular pool (diameter 80 cm and height 40 cm), and the pool was divided into four quadrants. A platform (diameter 8 cm) was placed in the middle of one quadrant and was submerged 1 cm below the water surface. In the training trials, the mice were placed randomly in the water facing the pool wall and allowed to find the hidden platform for 60 s. If the mice were unable to find the platform within 60 s, they were guided and allowed to stay on the platform for 15 s. The mice were given three trials per day for 3 consecutive days, and a probe trial of the MWM test was then performed. In the primary test, the experiment was performed as described earlier. For the secondary test, the pool was filled with transparent water, and the platform was placed in the target quadrant. The time for each mouse to reach the exposed platform was recorded. All trials were recorded and analyzed by SMART video tracking system (SMART v3.0, Panlab SL, Barcelona, Spain).

### 2.5. Measurement of MDA Generation

The MDA levels in the brain were determined using the method described by Ohkawa et al. [23]. After completion of the behavioral tests, the brain tissue was dissected and homogenized with 0.9% NaCl. The homogenate was mixed with 1% phosphoric acid and 0.67% thiobarbituric acid (Lancaster Synthesis, Ward Hill, MA, USA). This solution mixture was boiled for 45 min at 95 °C and placed in an ice bath for cooling. Next, 2 mL 1-butanol (Samchun Pure Chemical Co., Pyeongtaek, Korea) was added and then centrifuged at 3000× *g* rpm for 10 min. The absorbance values of the supernatant were measured using a microplate reader (Thermo Fisher Scientific, Vantaa, Finland) at 540 nm, and the level of lipid peroxidation was calculated using an MDA standard curve.

### 2.6. Measurement of NO Generation

The NO concentration in the brain was determined according to the method by Schmidt et al. [24]. Briefly, 150 μL brain tissue homogenate was mixed with 130 μL distilled water. Then, 20 μL mixed solution was added to an equal amount of phosphoric acid and 0.1% n-(1-naphthyl)ethylene-diamide dihydrochloride. The absorbance value of the mixture was measured at 540 nm using a microplate reader (Thermo Fisher Scientific, Vantaa, Finland). The NO production was calculated using a NaNO_2_ standard curve.

### 2.7. Western Blotting

The mice brains were homogenized with RIPA lysis buffer (50 mM Tris-HCl pH7.5, 0.1% SDS, 1% Triton X-100, 150 mM NaCl, 0.5% sodium deoxycholate, and 2 mM EDTA containing a protease inhibitor cocktail). The mixture was centrifuged (13,000× *g* rpm for 30 min), and the supernatants were collected. The protein concentrations were determined using the Bio-Rad protein assay kit (Bio-Rad, Hercules, CA, USA). Equal concentrations of the protein were separated in 10–13% sodium dodecyl sulfate polyacrylamide gel electrophoresis (SDS-PAGE) and then transferred to polyvinylidene fluoride membranes (Millipore, Billerica, MA, USA). The membranes were subsequently incubated with 10% skim milk for 60 min and treated with primary antibody at 4 °C overnight. The following primary antibodies were used: (Bax (1:200, Santa Cruz Biotechnology, Santa Cruz, CA, USA), Bcl-2 (1:200, Santa Cruz Biotechnology), cleaved caspase-3 (1:200, Santa Cruz Biotechnology), anti-APP C-terminal (1:1000; Sigma), presenilin 1 (PS1, 1:1000, Cell Signaling Technology, Beverly, MA, USA), PS2 (1:1000, Cell Signaling Technology), and β-actin (1:200, Santa Cruz)). The membranes were further incubated with secondary antibodies (1:1000, Cell Signaling) for 1 h, and protein bands were visualized by Davinci-Chemiluminescent imaging system (CoreBio, Seoul, Korea) using pico-enhanced peroxidase detection (ELPIS-Biotech, Daejeon, Korea). The intensity of the protein bands was quantified by Image J (National Institutes of Health, Bethesda, MD, USA).

### 2.8. Statistical Analysis

The statistical differences between the control and Aβ_25–35_, MFSCE-, PLP-, MP-, and DO-administered groups were determined by nonparametric one-way ANOVA followed by Duncan’s post-hoc test with the IMB SPSS statistics programs 23 (IBM Co., Armonk, NY, USA). In the T-maze and NOR tests, the difference in the perception abilities between the training and test sessions was ascertained using nonparametric Student’s *t*-test. Significance was set at *p* < 0.05.

## 3. Results

### 3.1. T-Maze Test

**Figure 2:**
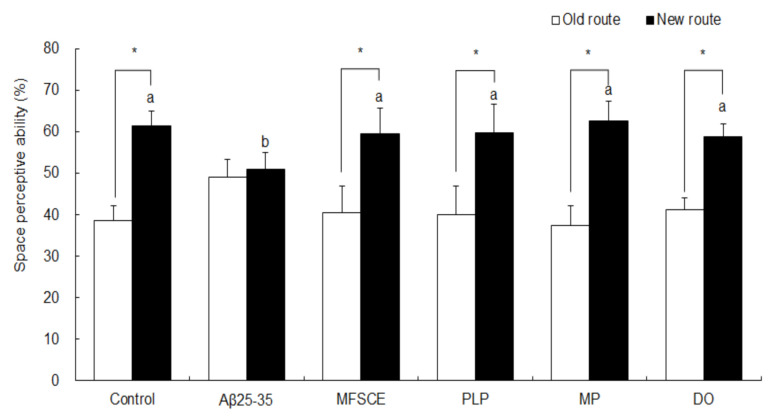
Effect of MFSCE on space perception ability using the T-maze test in Aβ_25–35_-injected mice. ^a,b^ Means with different letters are significantly different (*p* < 0.05) from each group in test session. Data are expressed as mean ± SD (*n* = 6). * The space perception abilities for the old and new routes are significantly different as analyzed by the Student’s *t*-test (*p* < 0.05). Control group: 0.9% NaCl saline i.c.v. + 0.9% NaCl saline i.p.; Aβ_25–35_ group: Aβ_25–35_ i.c.v. + 0.9% NaCl saline i.p.; MFSCE group: Aβ_25–35_ i.c.v. + MFSCE (100 mg/kg) i.p.; PLP group: Aβ_25–35_ i.c.v. + PLP (1 mg/kg) i.p.; MP group: Aβ_25–35_ i.c.v. + MFSCE (100 mg/kg) and PLP (1 mg/kg) i.p.; DO group (positive control): Aβ_25–35_ i.c.v. + donepezil (5 mg/kg) i.p. injection.

The effect of MFSCE on Aβ_25–35_-induced spatial memory impairment was measured using a T-maze test, 7 days after sample (MFSCE, PLP, or MP) treatment. As shown in Figure 2, the control group showed higher exploration and entries into the new route (61.4%) than to the old route (38.6%; Student’s *t*-test, *p* = 0.000), which were revealed in the training session. In comparison, no significant difference in the space perception ability between the old (49.1%) and new (50.9%) routes was observed in the Aβ_25–35_-injected mice (Student’s *t*-test, *p* = 0.461). However, MFSCE-, PL-, and MP-treated mice showed improved memory performance, as indicated by significantly increased entries into the new routes compared to the old route (40.6–59.4%, 40.1–59.9%, and 37.4–62.6%, respectively; Student’s *t*-test, *p* = 0.000). The DO group was used as a positive control, and DO treatment attenuated cognitive impairments in mice, which increased the ratio of new route entries compared to that of the old route (58.9 vs. 41.1%; Student’s *t*-test, *p* = 0.000).

### 3.2. Novel Object Recognition (NOR) Test

**Figure 3:**
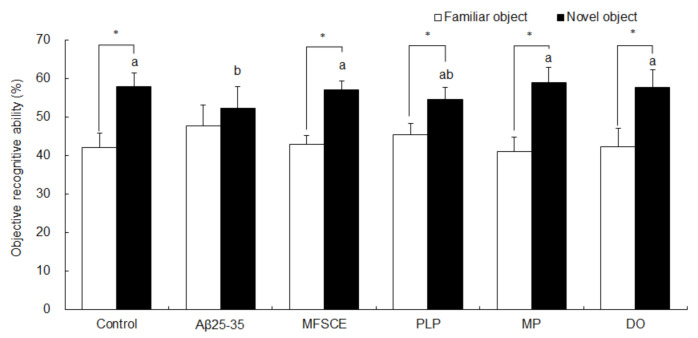
Effect of MFSCE on object perception ability by novel object recognition test in Aβ_25–35_-injected mice. ^a,b^ Means with different letters are significantly different (*p* < 0.05) from each group in the test session. Data are expressed as mean ± SD (*n* = 8). * The object perception abilities for familiar and novel objects differed significantly when analyzed by the Student’s *t*-test (*p* < 0.05). Control group: 0.9% NaCl saline i.c.v. + 0.9% NaCl saline i.p.; Aβ_25–35_ group: Aβ_25–35_ i.c.v. + 0.9% NaCl saline i.p.; MFSCE group: Aβ_25–35_ i.c.v. + MFSCE (100 mg/kg) i.p.; PLP group: Aβ_25–35_ i.c.v. + PLP (1 mg/kg) i.p.; MP group: Aβ_25–35_ i.c.v. + MFSCE (100 mg/kg) and PLP (1 mg/kg) i.p.; DO group (positive control): Aβ_25–35_ i.c.v.+ donepezil (5 mg/kg) i.p. injection.

To determine the ability of memory recognition, the NOR test was performed after the T-maze test. In the NOR test, mice were exposed to two identical objects, and one object was then replaced by a novel object. As illustrated in Figure 3, the control group of mice were able to discriminate between familiar and novel objects, showing an increase in time spent exploring novel object in relation to time spent exploring a familiar object (Student’s *t*-test, *p* = 0.000). However, Aβ_25–35_-injected mice exhibited no significant differences in preference for familiar or novel object (Student’s *t*-test, *p* = 0.109), demonstrating that mice injected with Aβ_25–35_ did not remember the object that they were exposed to in the previous trial. Compared to the Aβ_25–35_-injected group, treatment with MFSCE, PLP, MP, or DO induced a significantly higher preference for the novel object compared to a familiar object (Student’s *t*-test, *p* = 0.109), indicating that MFSCE, PLP, and MP prevented Aβ_25–35_-induced object perception impairment.

### 3.3. Morris Water Maze (MWM) Test

**Figure 4:**
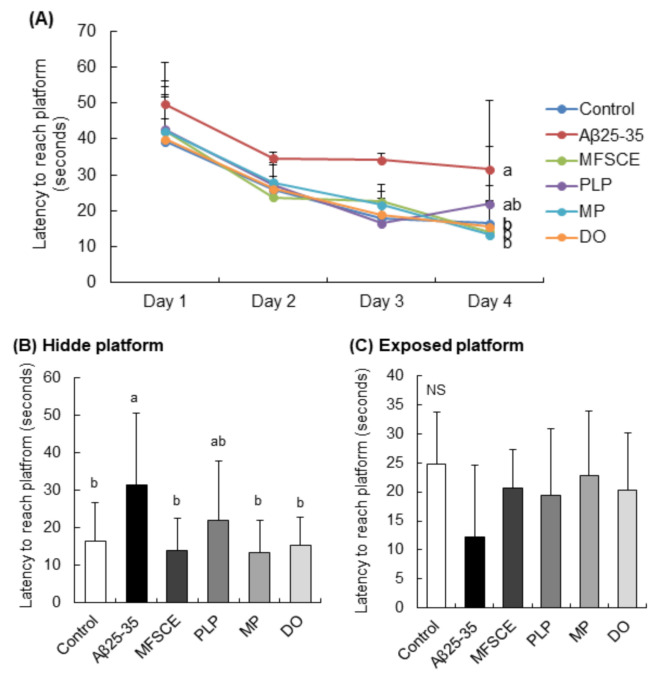
Effect of MFSCE on the spatial memory ability in the Morris water maze test in Aβ_25–35_-injected mice. (**A**) Effects of MFSCE on the escape latency to the platform during 4 days in the Morris water maze test in Aβ_25−35_-injected mice. (**B**) Effects of MFSCE on latency to reach the hidden platform. (**C**) Effects of MFSCE on the latency to reach the exposed platform. Data are expressed as mean ± SD (*n* = 10). ^a,b^ Means with different letters are significantly different (*p* < 0.05) from each other. NS: no significance. Control group: 0.9% NaCl saline i.c.v. + 0.9% NaCl saline i.p.; Aβ_25–35_ group: Aβ_25–35_ i.c.v. + 0.9% NaCl saline i.p.; MFSCE group: Aβ_25–35_ i.c.v. + MFSCE (100 mg/kg) i.p.; PLP group: Aβ_25–35_ i.c.v. + PLP (1 mg/kg) i.p.; MP group: Aβ_25–35_ i.c.v. + MFSCE (100 mg/kg) and PLP (1 mg/kg) i.p.; DO group (positive control): Aβ_25–35_ i.c.v.+ donepezil (5 mg/kg) i.p. injection.

The MWM test was conducted to assess the long-term and spatial learning and memory abilities. This behavioral test relies on the rodents’ motivation to escape water by searching for the hidden platform, which was placed using visually different cues in the wall of the pool. During the training session, the time spent by the control group searching for the hidden platform gradually decreased (Figure 4A). However, the Aβ_25–35_-injected mice group did not show this trend. The results of three trials for 3 days showed that treatment with MFSCE, MP, and DO significantly decreased the time taken to reach the target platform compared to only Aβ_25–35_-injected mice (Figure 4B; *F*_(5,53)_ = 3.068, *p* = 0.017). In addition, there was no statistically significant intergroup difference in the time taken to reach the exposed platform (Figure 4C; *F*_(5,53)_ = 1.131, *p* = 0.520), indicating that the swimming or visual ability was unaltered in mice. Based on these data, MFSCE and MP improved learning and memory capabilities in Aβ_25–35_-injected mice.

### 3.4. Effect of MFSCE on the Levels of Malondialdehyde (MDA) and Nitric Oxide (NO) in Aβ_25–35_-Injected Mice Brain

The antioxidant effect of MFSCE treatment against Aβ_25–35_-induced MDA and NO overproduction in the brain was elucidated. As shown in Table 2, MDA concentrations were significantly increased in the Aβ_25–35_-injected mice brain (108.11 nmol/mg protein) compared to the noninjected mice brain (74.05 nmol/mg protein). However, MDA concentrations were reduced by MFSCE injection (86.67 nmol/mg protein; *F*_(5,36)_ = 17.611, *p* = 0.000). In addition, Aβ_25–35_ injection significantly elevated the NO levels from 9.68 to 28.25 μmol/mg protein, whereas MFSCE inhibited NO generation to 14.33 μmol/mg protein. Therefore, MFSCE could attenuate the oxidative stress induced by Aβ_25–35_ in the brain of AD mice. Furthermore, compared to MFSCE- or PLP-only treatment, the MFSCE and PLP combination treatment significantly suppressed MDA and NO production to 80.43 and 11.43 μmol/mg protein, respectively (*F*_(5,36)_ = 25.317, *p* = 0.000). The significantly lower levels of MDA and NO in the brain of MP-treated mice indicated that the MFSCE and PLP combination could effectively attenuate Aβ_25–35_-induced oxidative damage.

### 3.5. Effect of MFSCE on Neuronal Apoptosis in Aβ_25–35_-Injected Mice Brain

**Figure 5:**
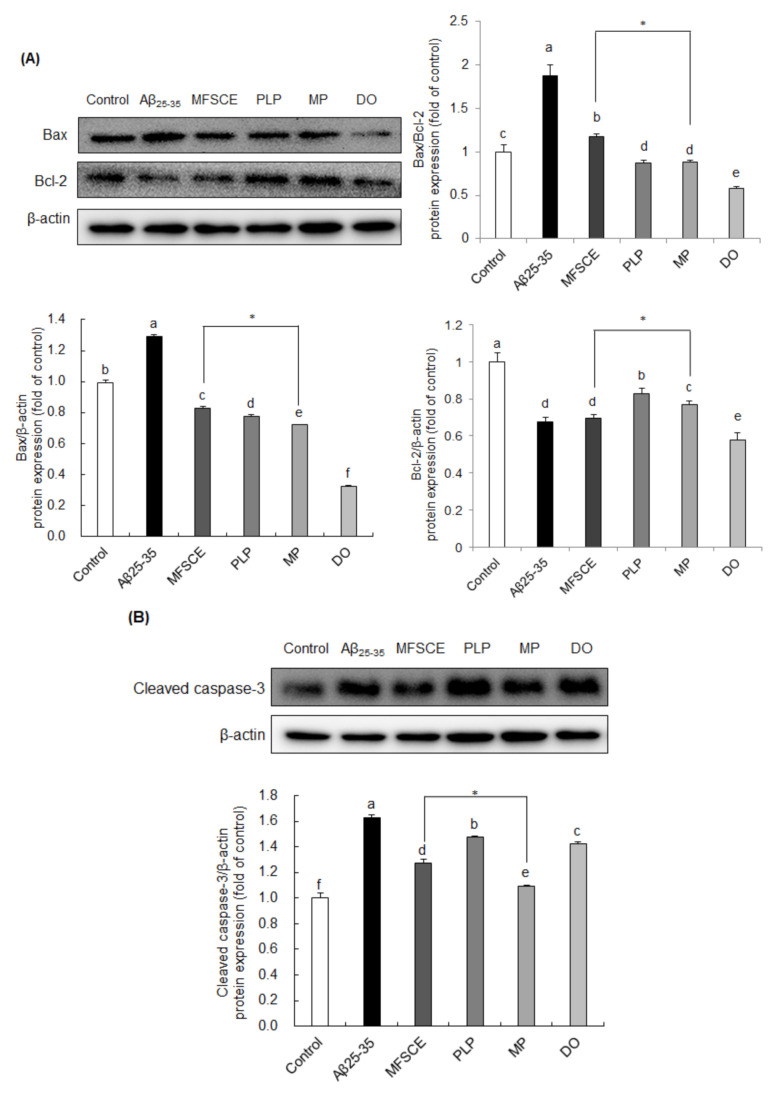
Effect of MFSCE on the neuronal-apoptosis-related protein expression in the brain of Aβ_25–35_-injected mice. Western blotting and quantitative analysis of (**A**) Bax/Bcl-2 ratio, Bax, and Bcl-2 and (**B**) cleaved caspase-3 protein expression levels in the brain. Data are expressed as mean ± SD (*n* = 3). ^a–e^ Means with different letters are significantly different (*p* < 0.05) from each group. * β-actin served as the loading control. Asterisk (*) indicates statistically significant differences (*p* < 0.05) between MFSCE and MP groups. *p* < 0.05 vs. MFSCE group. Control group: 0.9% NaCl saline i.c.v. + 0.9% NaCl saline i.p.; Aβ_25–35_ group: Aβ_25–35_ i.c.v. + 0.9% NaCl saline i.p.; MFSCE group: Aβ_25–35_ i.c.v. + MFSCE (100 mg/kg) i.p.; PLP group: Aβ_25–35_ i.c.v. + PLP (1 mg/kg) i.p.; MP group: Aβ_25–35_ i.c.v. + MFSCE (100 mg/kg) and PLP (1 mg/kg) i.p.; DO group (positive control): Aβ_25–35_ i.c.v.+ donepezil (5 mg/kg) i.p. injection.

To examine whether MFSCE has a protective effect on Aβ_25–35_-induced neuronal apoptosis, changes in the levels of Bax, Bcl-2, and cleaved caspase-3 were analyzed by Western blotting. As shown in Figure 5A, the Bax/Bcl-2 ratio was increased following Aβ_25–35_ injection, whereas MFSCE, PLP, and MP treatment significantly reversed the changes in the Bax/Bcl-2 ratio. In particular, MP treatment markedly downregulated Bax and upregulated Bcl-2 protein levels compared to MFSCE treatment, leading to reduced Bax/Bcl-2 expression. To further confirm the role of MFSCE in caspase activation, we analyzed the protein expression of cleaved caspase-3 (Figure 5B). Levels of cleaved caspase-3 were enhanced in the Aβ_25–35_-injected brain compared to noninjected mice, whereas treatment with MFSCE inhibited caspase-3 activation, further indicating that MFSCE may have antiapoptotic effects induced by Aβ_25–35_. In addition, the combination treatment of MFSCE and PLP effectively suppressed caspase-3 cleavage. These results suggest that the beneficial effect of MFSCE on learning and memory improvement could correlate with its antiapoptotic properties and that these effects may be enhanced by PLP.

### 3.6. Effect of MFSCE on the Amyloidogenic Pathway in Aβ_25–35_-Injected Mice Brain

**Figure 6:**
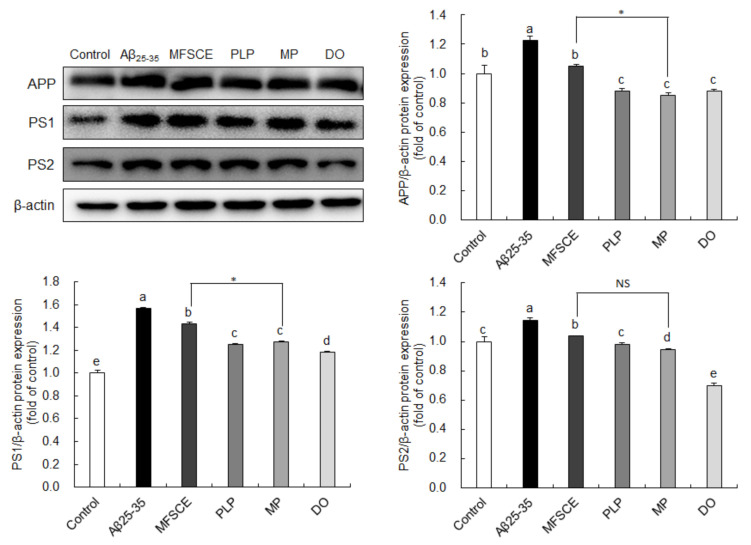
Effect of MFSCE on the amyloidogenic-pathway-related protein expression in the brain of Aβ_25–35_-injected mice. Western blotting and quantitative analysis of APP, PS1, and PS2 protein expression levels in the brain. Data are expressed as mean ± SD (*n* = 3). ^a–e^ Means with different letters are significantly different (*p* < 0.05) from each group. Asterisk (*) indicates statistically significant differences (*p* < 0.05) between MFSCE and MP groups. β-actin served as the loading control. Control group: 0.9% NaCl saline i.c.v. + 0.9% NaCl saline i.p.; Aβ_25–35_ group: Aβ_25–35_ i.c.v. + 0.9% NaCl saline i.p.; MFSCE group: Aβ_25–35_ i.c.v. + MFSCE (100 mg/kg) i.p.; PLP group: Aβ_25–35_ i.c.v. + PLP (1 mg/kg) i.p.; MP group: Aβ_25–35_ i.c.v. + MFSCE (100 mg/kg) and PLP (1 mg/kg) i.p.; DO group (positive control): Aβ_25–35_ i.c.v.+ donepezil (5 mg/kg) i.p. injection.

To determine whether the observed improvement of learning and memory function and antiapoptotic effect are associated with APP processing, we analyzed APP, PS1, and PS2 protein expression levels by Western blotting. As shown in Figure 6, significant increases in APP protein levels were observed in the Aβ_25–35_-injected group compared to the control group, whereas PLP and MP significantly downregulated APP protein expression. In addition, PS1 and PS2 expressions were significantly higher in the Aβ_25–35_ groups compared to the control group. However, PS1 and PS2 expressions in the brain were significantly reduced by treatment with PLP and MP. In particular, MP showed considerably lower APP and PS1 protein levels in the brain compared to treatment with MFSCE or PLP only. These findings further indicate that the combination of MFSCE and PLP may prevent the progression of AD pathology by inhibiting the amyloidogenic pathway.

## 4. Discussion

Stem-cell therapy has been established as an effective regenerative therapy for brain function in animal models of neurodegenerative diseases, such as Parkinson’s disease, stroke, and AD [25,26,27]. Stem cells derived from neural, bone marrow, and adipose tissues have been observed to alleviate Aβ deposition and improve cognitive impairment in AD mouse models [28,29]. Specifically, adipose-tissue-derived stem cells (ADSCs) have been proven to be safe, capable of self-renewal, and have the potential to differentiate into adipocytes, chondrocytes, osteoblasts, and neurocytes [30]. Furthermore, these cells are easily accessible and show high proliferation rates with lower senescence ratios in vitro compared to the rates in other stem cells [31]. However, stem-cell-based therapeutics is associated with several problems, such as the difficulty in preservation or transport of stem cells for administration to patients. Membrane-free stem cells could overcome the limitation of cell-based therapy. However, the protective effect of MFSCE and their mechanism of action in AD have not yet been studied. This study evaluated the therapeutic effect of MFSCE on Aβ-induced learning and memory impairment and determined the mechanism of action of MFSCE.

Experimental intracerebroventricular infusion of Aβ peptides can mimic AD models [32]. Injection of Aβ causes learning and memory deficits as well as behavioral alterations, which have been observed in AD patients [33,34]. Compared to Aβ_1–42_, Aβ_25–35_ induces neurotoxicity more rapidly, leading to more oxidative damage [35]. To examine the protective effect of MFSCE on Aβ_25–35_-induced learning and memory impairment, the T-maze, NOR, and MWM tests were carried out. The T-maze test was performed to evaluate the ability of mice to enter new routes that they had not entered in previous trials. The results of the T-maze test indicated no significant difference in the alternation behavior between the old or new routes in Aβ_25–35_-injected mice. A similar trend was observed in NOR tests. The group of Aβ_25–35_-injected mice had no significant difference in the exploration time for familiar object and novel object, indicating that AD mice were unable to recognize the new route/novel object. However, the mice in the MFSCE, PLP, and MP groups spent more time exploring the new route/novel object than they expended on the old route/familiar object. These findings suggest that MFSCE may improve cognitive ability against Aβ_25–35_ in a mouse model. Next, we conducted the MWM test to confirm whether MFSCE improves long-term memory ability in Aβ_25–35_-injected AD mice. In our findings, Aβ_25–35_-injected mice took significantly longer to reach the hidden platform, demonstrating impaired spatial memory ability. In contrast, in mice treated with MFSCE and MP, the time taken to reach the platform decreased significantly, indicating that MFSCE and MP can protect against the long-term memory impairment indued by Aβ_25–35._ An earlier study found that learning and memory impairment improved after ADSC injection in AD mice [20]. The results of the present study indicate that MFSCE derived from adipose tissue could prevent learning and memory deficits induced by Aβ_25–35_.

Aβ-induced cognitive deficit correlates with ROS/RNA production, lipid peroxidation, and antioxidant enzyme inactivation, resulting in neuronal damage [36,37]. Montine et al. [38] demonstrated that increasing levels of oxidative stress markers, such as ROS, RNA, and MDA, were observed in the neurons surrounding Aβ plaques in AD patients. Therefore, we measured MDA and NO levels in the brain of mice. The findings of our study revealed that Aβ_25–35_ injection significantly elevated MDA and NO levels compared to the levels in noninjected mice. However, MFSCE, PLP, and MP administration significantly reduced Aβ_25–35_-induced MDA and NO production in the brain, and no significant differences in MDA and NO levels were detected between the treatment groups and the DO group. ADSCs exert an antioxidant function by regulating the activity of superoxide dismutase, which is an antioxidant enzyme [39]. Furthermore, the injection of ADSCs effectively reduced lipid peroxidation following acute kidney injury in rats [40], while PLP significantly inhibited the oxidative stress induced by the Cu^II^–Aβ complex [41]. Therefore, the antioxidant properties of MFSC, PLP, and MP are attributed to their protective effect against Aβ_25–35_-induced cognitive impairment by inhibition of lipid peroxidation and scavenging of NO.

Numerous studies have demonstrated the involvement of neuronal apoptosis in AD [42,43,44]. The Aβ within the brain can induce neurotoxic substances, including cytokines, ROS, and RNS, which ultimately result in neuronal cell death [45,46,47]. The Bcl-2 family plays a crucial role in the mitochondrial apoptosis pathway, and they are composed of the antiapoptotic protein Bcl-2 and the proapoptotic protein Bax. Furthermore, Bax overexpression following activation of the apoptotic signaling pathway and subsequent release of cytochrome C from the mitochondria into the cytosol induces caspase activation and cell death [48]. However, Bcl-2 inhibits the Bax-mediated apoptotic signaling pathway and promotes cell survival [49,50]. Caspase-3 is the eventual protein in the apoptotic cascade and is cleaved by mitochondrial cytochrome C [51]. Marin et al. [52] reported that APP can be directly cleaved by caspase during neuronal apoptosis and leads to the formation of Aβ. Results from our previous study demonstrated that MFSCE treatment significantly reduced the Bax/Bcl-2 ratio in oxidative-stress-induced LLC-PK_1_ cells [11]. Moreover, MFSCE decreased Bax/Bcl-2 protein expression in Aβ_25–35_-induced SH-SY5Y [13]. To clarify the protective mechanism of MFSCE action on Aβ_25–35_-induced cognitive impairment, the expressions of apoptosis-related protein were analyzed by Western blotting in the brain of mice. In the present study, the Bax/Bcl-2 ratio and the protein levels of cleaved caspase-3 were higher in the Aβ_25–35_-injected mice group compared to the levels in the noninjected mice group. Although MFSCE effectively downregulated the expressions of the abovementioned proteins, the expression of cleaved caspase-3 was more effectively suppressed by MP treatment, suggesting a potentiating benefit of the MFSCE and PLP combination. Therefore, these results indicate that MP ameliorates Aβ_25–35_-induced cognitive impairment and oxidative stress in the brain of mice, at least partly, through the regulation of the caspase-3-dependent apoptotic pathway.

Previous studies on Aβ injection have reported the overexpression of APP and amyloidogenic-pathway-related proteins, such as BACE, PS1, and PS2 [53,54,55]. Therefore, we further investigated whether these protective mechanisms of MFSCE on oxidative stress, neuronal apoptosis, and cognitive dysfunction are mediated through modulation of the amyloidogenic pathway. We observed that APP, PS1, and PS2 protein levels were significantly upregulated by Aβ_25–35_ injection, indicating that Aβ_25–35_ could induce the amyloidogenic pathway via γ-secretase activity. However, treatment with MFSCE, PLP, or MP reduced these protein expressions. Our previous study revealed that MFSCE effectively decreased APP, BACE, PS1, and PS2 protein expression against Aβ_25–35_ in SH-SY5Y neuronal cells [13]. Furthermore, MP induced considerably lower APP, PS1, and PS2 protein expression than that observed in the group treated with MFSCE alone. Thus, the improved cognitive function and inhibition of neuronal apoptosis are consistent with the downregulation of the amyloidogenic pathway, and combination therapy with MFSCE and PLP could enhance their inhibitory effects on Aβ production. The γ-secretase activating protein (GSAP) that facilitates Aβ production is mediated by caspase-3 [56]. Blockade of caspase-3 activation resulted in a reduction in GSAP levels and Aβ formation in mice. This evidence indicates that the downregulation of PS1 and PS2 by MP treatment may be attributed to the inhibition of caspase-3 activation.

Vitamin B_6_ plays an important role as a cofactor in general cellular metabolism and mediates the biosynthesis of amino acids and fatty acids as well as neurotransmitters and organelle-specific compounds [17]. In addition, vitamin B_6_ has been shown to have a critical role in antioxidant and anti-inflammatory responses and possibly even in immune responses and brain function [57,58,59]. Geng et al. [60] reported that PLP, a biologically active form of vitamin B_6_, affects the brain developmental process by participating in the synthesis of protein, carbohydrates, lipids, and nucleic acid [61,62]. In addition, vitamin B_6_ is a water-soluble vitamin, which might be safer than lipid-soluble vitamins and more readily available in clinical application. Consistent with this evidence, our results suggest that PLP may promote neuronal survival rate and support MFSCE metabolism through the synthesis of biomolecules in the brain. Although it is uncertain whether PLP directly influences the protective effect of MFSCE on cognitive improvement in mice, a potential mechanism of action has been proposed to explain the biological correlation of PLP with MFSCE in this study.

## 5. Conclusions

In conclusion, the injection of Aβ_25–35_ induced APP processing and subsequently oxidative stress and neuronal apoptosis, which resulted in behavioral dysfunction in mice. However, MFSCE reversed the Aβ_25–35_-induced learning and memory impairment by suppressing oxidative stress as well as downregulation of apoptosis- and amyloidogenic-pathway-related protein markers in the brain. In addition, MP induced greater reduction in the protein expression of cleaved caspase-3, APP, PS1, and PS2 compared to MFSCE- or PLP-only treatment, suggesting that a combination of MFSCE and PLP may support MFSCE metabolism in the brain through a synergistic effect.

## Figures and Tables

**Table 1:** Mice groups and treatment protocol.

Group	n	Treatment
Control	10	0.9% NaCl saline i.c.v. + 0.9% NaCl saline i.p.
Aβ_25–35_	10	Aβ_25–35_ i.c.v. + 0.9% NaCl saline i.p.
MFSCE	10	Aβ_25–35_ i.c.v. + MFSCE (100 mg/kg) i.p.
PLP	10	Aβ_25–35_ i.c.v. + PLP (1 mg/kg) i.p.
MP	10	Aβ_25–35_ i.c.v. + MFSCE (100 mg/kg) and PLP (1 mg/kg) i.p.
DO	9	Aβ_25–35_ i.c.v. + donepezil (5 mg/kg) i.p.

**Table 2:** Effect of MFSCE on lipid peroxidation and NO generation in the brain of Aβ_25–35_-injected mice.

Group	Concentration	
	MDA (μmol/mg Protein)	NO (nmol/mg Protein)
Control	74.05 ± 4.39 ^d^	9.68 ± 1.07 ^d^
Aβ_25–35_	108.11 ± 7.47 ^a^	28.20 ± 6.56 ^a^
MFSCE	86.67 ± 8.64 ^bc^	14.33 ± 1.65 ^bc^
PLP	86.22 ± 2.20 ^b^	15.48 ± 1.88 ^b^
MP	80.43 ± 8.34 ^cd^	11.43 ± 3.15 ^cd^
DO	89.49 ± 9.57 ^bc^	15.25 ± 3.21 ^bc^

Data are expressed as mean ± SD (*n* = 7). ^a–d^ Means with different letters are significantly different (*p* < 0.05) from each group. Control group: 0.9% NaCl saline i.c.v. + 0.9% NaCl saline i.p.; Aβ_25–35_ group: Aβ_25–35_ i.c.v. + 0.9% NaCl saline i.p.; MFSCE group: Aβ_25–35_ i.c.v. + MFSCE (100 mg/kg) i.p.; PLP group: Aβ_25–35_ i.c.v. + PLP (1 mg/kg) i.p.; MP group: Aβ_25–35_ i.c.v. + MFSCE (100 mg/kg) and PLP (1 mg/kg) i.p.; DO group (positive control): Aβ_25–35_ i.c.v.+ donepezil (5 mg/kg) i.p. injection.

## Data Availability

The data associated with this research are available and can be obtained by contacting the corresponding author.
